# Modified McKissock's Breast Reduction Technique: A Case Series and Description of Our Technique Modification

**DOI:** 10.1016/j.jpra.2023.10.017

**Published:** 2023-11-03

**Authors:** Hatan Mortada, Eman A. Ibrahim, Taghreed R. Alhumsi

**Affiliations:** a Division of Plastic Surgery, Department of Surgery, King Saud University Medical City, King Saud University and Department of Plastic Surgery & Burn Unit, King Saud Medical City, Riyadh, Saudi Arabia; b Private Practice, Riyadh, Saudi Arabia

**Keywords:** Breast reduction, McKissock's technique, Super and inferior vascular pedicles, Nipple and areola complex preservation, Complications, Aesthetic outcomes

## Abstract

**Background:**

The modified McKissock breast reduction technique uses upper and lower vascular pedicles to reduce breast size and reshape the breasts. This technique has gained significant interest in recent years because of its potential to minimize surgical complications. The current study aims to report our experience and results with our refined version of the McKissock technique.

**Methods:**

We conducted a prospective cohort study on patients with breast hypertrophy between 2022 and 2023 to evaluate the modified McKissock breast reduction technique. Two main alterations were made to the original McKissock technique. First, the superior pedicle was modified to create a superomedial pedicle. Second, the inferior pedicle was thinned to form a dermoseptal pedicle with a 4 cm wide base.

**Results:**

A total of 13 patients underwent surgery using the modified McKissock breast reduction technique. The average age of the patients was 37.2 years. For the right breast, the weight of tissue resected during reduction ranged from 189 g to 695 g (average 379 g). For the left breast, the resection weight range was 160 g to 608 g (average 370 g). There were no complications except one patient who developed partial nipple necrosis on the left side. All patients expressed satisfaction with the outcomes.

**Conclusion:**

Our modified McKissock breast reduction technique shows promise as a method for reducing breast size. It offers several potential advantages, including improved preservation of the nipple and areola complex, more precise breast shaping, contouring capabilities, and reduced risk of complications. Although the early results of this technique are encouraging, further research is required to evaluate its long-term benefits and risks fully.

## Introduction

Breast hypertrophy, or macromastia, is characterized by abnormally large breast size.^1^ It may impact the patient physically and psychologically—chronic pain, skin irritation, and impaired mobility.^2^ As a result, breast reduction surgery is a common treatment approach, and several techniques have been developed.^1^ However, certain limitations highlight the need for innovative surgical methods to improve outcomes.^3^^,^^4^

Various breast reduction techniques are available, each with advantages and disadvantages. They aim to remove tissue appropriately and symmetrically while preserving nipple-areola complex (NAC) viability and function for good long-term aesthetic outcomes. Thus, new modifications continue to evolve.^1^, ^2^, ^3^, ^4^, ^5^ The optimal technique depends on factors like breast size, ptosis, and the surgeon's expertise.^6^

The McKissock technique uses a well-vascularized dermoglandular bipedicle to reposition the NAC in large reductions.^7^ However, concerns exist regarding inadequate projection and bottoming out over time.^8^^,^^9^ Modifications, including pedicle beveling, S-shaped upper pole folding, and dermal suspension, aim to address these limitations.^10^^,^^11^

Our study details modifications to the McKissock technique using superomedial and thinned inferior pedicles. We aim to present our modified approach and provide a basis for further research. By sharing our results, we hope to contribute to future advancements in breast reduction surgery.

## Methods and Materials

### Patient selection and study design

This prospective cohort study included 13 patients with breast hypertrophy who underwent breast reduction surgery at a private center in Riyadh, Saudi Arabia. Patients were selected based on their willingness to participate and suitability for the modified bipedicled technique performed by the senior surgeon (T.A.). We excluded patients lost to follow-up or without outcome assessments. Exclusion criteria were high anesthesia risk with American Society of Anesthesiology score ≥3, organ failure, diabetes mellitus, collagen vascular disease, and bleeding disorders. The primary outcome was the complication rate. Secondary outcomes included demographics and weight of resected breast tissue.

### Data collection

We developed a data collection sheet based on variables used in previous studies with similar objectives. Preoperative data collected comprised age, body mass index (BMI), and breast measurements. Postoperative data, including breast size, shape, and complications, were gathered at 1 month and 6 months follow-up.

### Ethical consideration

This study was conducted following the Declaration of Helsinki after obtaining ethical approval from the Institutional Review Board. We adhered to the STROBE guidelines for conducting and reporting this prospective cohort study.^12^ All methods complied with relevant regulations and guidelines. Written informed consent was obtained from all patients prior to participation. Patient confidentiality was maintained throughout the study.

### Surgical technique description

Breast hypertrophy diagnosis was based on a comprehensive medical history and physical examination. Preoperative blood tests obtained a baseline for complete blood count, creatinine, and electrolytes concentrations. The senior plastic surgeon (T.A.) performed all reductions using the described technique, with adjustments as needed per patient. All patients provided informed consent after discussing risks, benefits, and potential complications with the provider. General anesthesia with endotracheal intubation was administered in all cases, along with intravenous antibiotics at induction. Patients were positioned supine, and surgical preparation and drapes were applied as standard for breast reduction.

**Figure 1:**
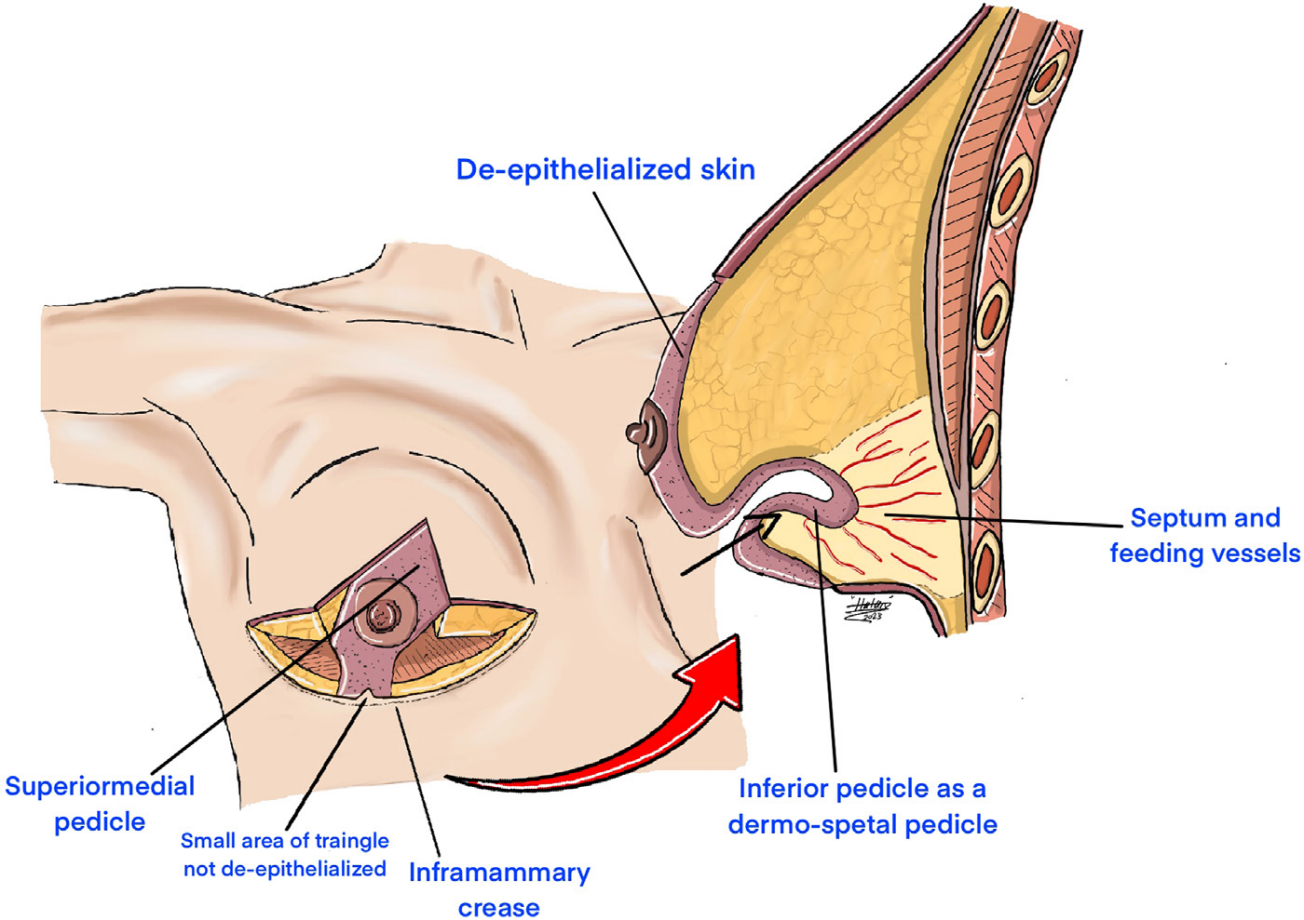
An illustration of our modified McKissock's technique, including a superomedial pedicle and a thinned inferior pedicle as dermoseptal pedicles

**Figure 2:**
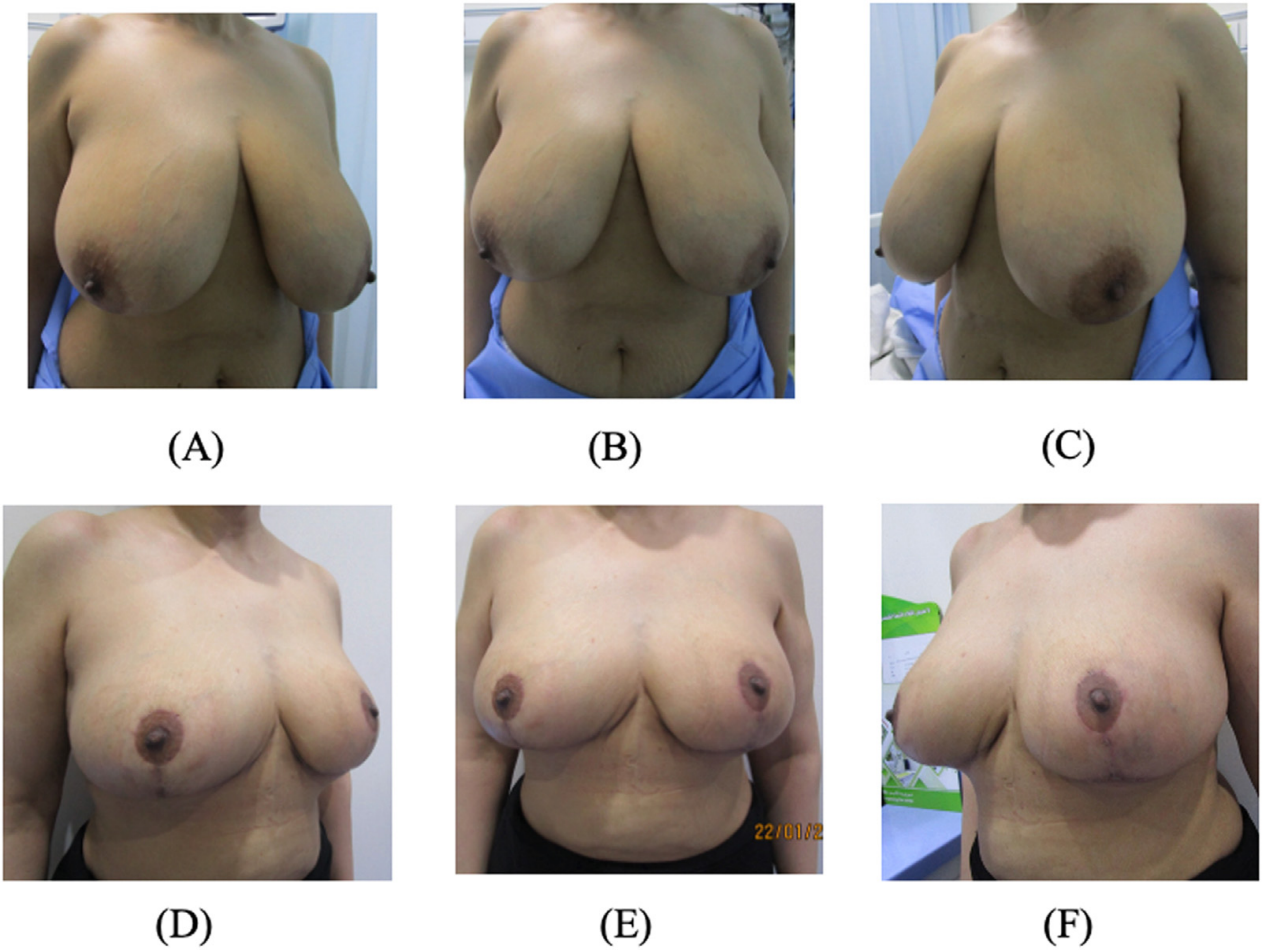
The steps followed to do the of the super and inferior vascular pedicles in breast reduction surgery.

This is a bipedicled technique combining superomedial and modified inferior pedicles. The superomedial pedicle was dissected and de-epithelialized as usual. The inferior pedicle was created by keeping a 4 cm width and drawing a 0.5 cm triangle from the inframammary fold base, which was not de-epithelialized to minimize necrosis at the inferior T-junction. After marking and de-epithelializing the inferior pedicle, the medial and lateral aspects were dissected, thinning it to a dermoseptal pedicle containing only septum and vessels. Figure 1 illustrates the approach. Postoperative dressings were applied, and patients were discharged same day with oral antibiotics and analgesics. Elevated head positioning, bending restrictions, and lateral sleep were recommended. Nipple-areolar complex viability was evaluated daily until postoperative day 5. Patients were permitted to shower the day after surgery. The technique is detailed in Figure 2.

## Results

**Figure 3:**
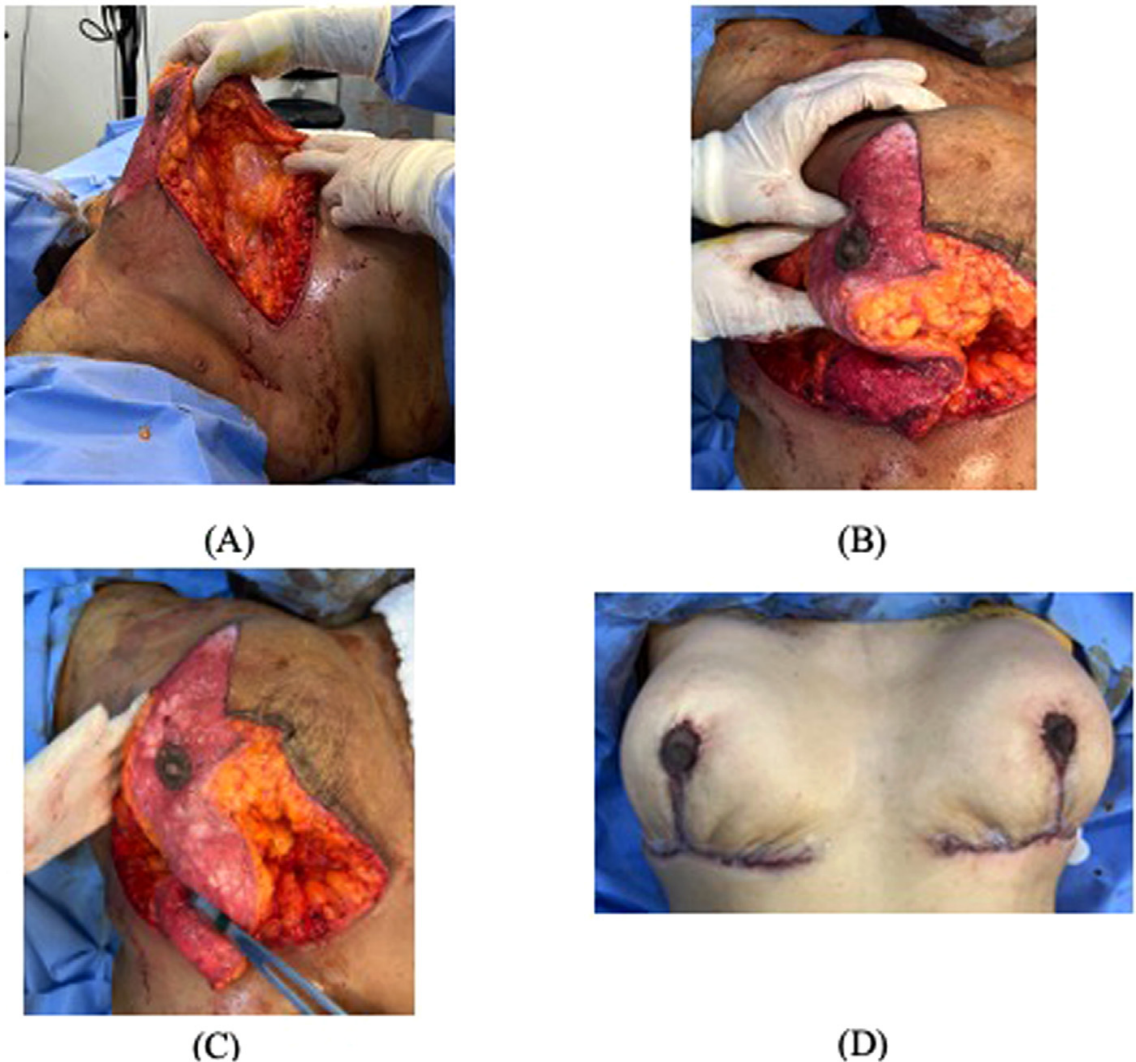
This figure shows the preoperative images (A-C) and 6-month postoperative images (D-F) of one of the patients who underwent breast reduction surgery using the modified McKissock technique.

Thirteen patients (26 breasts) underwent reduction using our modified technique for severe breast hypertrophy. Table 1 presents patient demographics. Mean age was 37.2 years (range 18-55 years). Average BMI was 27.45 kg/m^2^ (range 23.7-32.8 kg/m^2^). Preoperative hemoglobin concentration ranged from 11-14.7 g/dL (mean 13.2 g/dL). Right breast nipple-to-suprasternal notch distances were 27.5-36 cm. Left were 25-36 cm. For the right breast, resection weights ranged from 189 to 695 g (mean 379 g). Left breast ranged 160-608 g (mean 370 g). There were no complications except one patient who developed left partial nipple necrosis, treated conservatively with resolution. The mean operative time for our technique was approximately 163.15 minutes (range of 111-335 minutes). Figure 3 shows a sample case.

**Table 1:** Distribution of categorical variables of the included patients.

Patient	Age (Y)	Hgb (g/dL)	Height (m)	Weight (kg)	BMI	Measurement of right breast (SN-N)/ (N-IMF)	Measurement of left breast (SN-N)/ (N-IMF)	Weight of right breast (g)	Weight of left breast (g)	Complications	Operative time (mins)
1	38	11.1	1.59	66	26.1	30/15	31/16	423	430	Nil	148
2	18	12.4	1.49	54	24.3	33/15	31/15	347	325	Nil	133
3	35	12.51	1.56	70	28.7	32/13	32/13	254	244	Nil	153
4	25	12.6	1.57	63	25.5	29.5/13	30/14	298	304	Nil	148
5	41	12.7	1.56	78	32	31/13	30/ 12	333	458	Nil	139
6	55	13.1	1.48	72	32.8	36/14	35/12	657	579	Nil	115
7	27	13.5	1.63	63	23.7	26/12	25/12	195	185	Partial Nipple necrosis	111
8	39	13.8	1.63	79	29.7	33/18	34/17	543	562	Nil	173
9	49	14	1.52	65	28	31/13.5	31/14	440	450	Nil	167
10	30	14.2	1.51	57	24.9	27.5/12	28.5/13	189	166	Nil	142
11	36	14.7	1.67	80	28.6	37/17	36/17	695	608	Nil	355
12	49	14	1.52	65	28.1	31/13.5	31/14	440	450	Nil	150
13	55	13.1	1.48	72	32.8	36/14	35/12	657	579	Nil	187

(SN-N): Suprasternal notch to nipple distance; (N-IMF) Nipple–inframammary fold distance

## Discussion

Breast reduction surgery is commonly performed to alleviate physical and psychological consequences associated with large breast size.^2^ Traditional techniques remove excess breast tissue and skin, which can lead to loss of nipple sensation and decreased blood supply to the remaining tissue.^3^ A technique utilizing superior and inferior vascular pedicles was developed to address these limitations. In the present study, 13 patients underwent breast reduction using a technique described by the senior author, T.A., which involves the superomedial pedicle and a part of the inferior pedicle to reduce breast size. The mean BMI in our study was 27.45 kg/m^2^. Resection weights ranged from 189 to 695 g (mean 379 g) for the right breast and 160 to 608 g (mean 370 g) for the left. We describe modifications to the McKissock breast reduction technique using a superomedial and thinned inferior pedicle. We aim to present this modified approach and establish the groundwork for further research. Sharing our results, we hope to contribute to future breast reduction surgery techniques and research advancements.

The bipedicled technique utilizes two vascularized pedicles to supply blood flow to the breast tissue.^4^ Preserving these pedicles during reduction surgery helps maintain blood supply to the remaining breast tissue. This can reduce the risks of complications like nipple necrosis and shape deformities.^5^ The dual pedicle approach also enables greater flexibility in breast reshaping, as the tissue can be sculpted without compromising viability. The pedicles allow tissue to be moved and contoured to achieve a natural, aesthetic shape. Studies demonstrate this pedicle technique can decrease complications and improve patient satisfaction with breast reductions compared to other methods.^3^, ^4^, ^5^, ^6^ Our results align with these findings, as our modified McKissock reduction technique incorporating superomedial and inferior pedicles preserved nipple circulation. We observed only one case of minor necrosis among 13 patients, which resolved with conservative management. All patients were satisfied with their outcomes. By refining the pedicle design, we could sculpt the ideal breast shape for each patient while maintaining vascularity.

Excessive pedicle bulk and folding are common challenges in breast reduction that can lead to unaesthetic shapes and vascular kinking with risks of complications.^7^^,^^11^^,^^12^ Traditional pedicle techniques often create boxy, square breast contours, and pedicle redundancy when attempting to maintain circulation. Our modifications addressed this by thinning the inferior pedicle, ensuring blood supply without excessive volume or folding. This allowed more natural, aesthetic shaping while upholding viability. Innovations like Hinderer's dermal suspension for lifting and supporting the breast gland can be adapted to provide reinforcement in reduction techniques.^10^ Combining strategic pedicle thinning with suspensions can optimize shaping outcomes. Further developments in tissue engineering and biomaterials may provide additional solutions to balance vascularity and ideal form in large reductions. Our study demonstrates that with careful pedicle modification and mobilization, the benefits of a pedicled approach can be upheld while overcoming bulky shapes and sharp angles. However, long-term comparative data is limited, and 3D imaging would better quantify breast contour outcomes using varied pedicle designs. Larger-scale trials should examine shaping capabilities across reduction techniques. Though early results are promising, ongoing refinements in pedicle optimization will be key to maximizing both the art and science of breast reduction surgery. Our study achieved a mean operative time of 163.15 minutes (range 111-335 minutes) using the modified McKissock technique, compared with 180 minutes reported by Fischer et al. using traditional techniques in their 2014 study.^13^ Our technique's potential reduction in operative time may relate to modifications such as the pedicle design. However, given the limited sample size and overlap in operative time ranges between studies, definitive conclusions cannot be drawn. Larger-scale controlled trials are needed to validate if our modifications significantly reduce operative time by comparing them with existing breast reduction methods.

Dermal suspension of the bipedicle during reduction helps prevent a flat, deflated appearance postoperatively. It also reduces tension on medial and lateral skin flaps, improving healing and scarring. Comparisons of McKissock reductions with and without suspension show it limits bottoming out and maintains shape long-term. Our modified technique offers advantages that can enhance outcomes if properly implemented. However, specialized training in this approach is advisable to maximize benefits. Although early results are promising, multicenter studies should further compare variants in surgical technique, pedicle design, suspension methods, and long-term outcomes. Ongoing education and research will be key to optimizing safety, aesthetics, and patient satisfaction. Our study demonstrates initial feasibility of modifications to the McKissock breast reduction technique using strategic pedicle thinning and mobilization. This paves the way for future large-scale evaluations of evolving surgical innovations. With diligent pedicle optimization, the ideal balance of form and function may be achieved in reduction mammoplasty.

### Limitations and future recommendations

Although promising, this technique has limitations. It may not suit all patients, especially those with extremely large or dense breasts. It may prolong surgery time and increase costs compared with traditional reductions. Long-term outcomes warrant further research, including cosmetic results, risks, satisfaction, and quality of life. Hence, developing new technologies to optimize safety, efficacy, and accessibility will be important. We recommend future studies to examine surgical time metrics to evaluate the impact of a dual pedicle approach on efficiency. Larger sample sizes would also provide higher-quality evidence. More comparative data is needed on the trade-offs of different pedicle techniques in reduction mammoplasty. Although our early findings are encouraging, ongoing refinements and rigorous evaluation will determine how this modified McKissock approach may contribute to the evolution of breast reduction surgery.

## Conclusion

The combined use of superomedial and inferior pedicles in reduction mammoplasty provides multiple benefits. The dual pedicle design enhances nipple vascularity, supports wound healing, maintains projection, and enables excellent cosmetic results. The straightforward technique makes this a potentially valuable approach for breast hypertrophy that warrants ongoing evaluation. Although our initial outcomes are positive, larger-scale cohort studies with long-term follow-up are necessary to validate these preliminary findings further and compare this method with established techniques. With continued research to refine and optimize pedicle utilization, this modified McKissock reduction may represent a simple yet significant advancement for the field.

## References

[1] Hansen J, Chang S. Overview of breast reduction. UpToDate. Post TW ed. Waltham, MA: UpToDate Inc. 2018.

[2] Lapid O, de Groof EJ, Corion LU, Smeulders MJ, van der Horst CM. (2013). The effect of breast hypertrophy on patient posture. Arch Plast Surg.

[3] Andrades P, Prado A. (2008). Understanding modern breast reduction techniques with a simplified approach. J Plast Reconstr Aesthet Surg.

[4] Purohit S. (2008). Reduction mammoplasty. Indian J Plast Surg.

[5] Chiummariello S, Angelisanti M, Arleo S, Alfano C. (2013). Evaluation of the sensitivity after reduction mammoplasty. Our experience and review of the literature. Ann Ital Chir.

[6] Marouf A, Mortada H, Almutairi K. (2022). Preferences of different breast reduction techniques: Survey of board-certified plastic surgeons. Niger J Clin Pract.

[7] McKissock PK. (1972). Reduction mammaplasty with a vertical dermal flap. Plast Reconstr Surg.

[8] Ramon Y, Sharony Z, Moscona RA, Ullmann Y, Peled IJ (2000). Evaluation and comparison of aesthetic results and patient satisfaction with bilateral breast reduction using the inferior pedicle and McKissock’s vertical bipedicle dermal flap techniques. Plast Reconstr Surg.

[9] Hirshowitz B, Moscona AR. (1982). Modification of the bipedicled vertical dermal flap technique in reduction mammaplasty. Ann Plast Surg.

[10] Hinderer UT. (1978). Mammaplasty: the dermal brassière technique. Aesth Plast Surg.

[11] McCulley SJ, Macmillan RD. (2005). Planning and use of therapeutic mammoplasty—Nottingham approach. Br J Plast Surg.

[12] Von Elm E, Altman DG, Egger M, Pocock SJ, Gøtzsche PC, Vandenbroucke JP. (2007). The Strengthening the Reporting of Observational Studies in Epidemiology (STROBE) statement: guidelines for reporting observational studies. Lancet.

[13] Fischer JP, Cleveland EC, Shang EK, Nelson JA, Serletti JM. (2014). Complications following reduction mammaplasty: a review of 3538 cases from the 2005-2010 NSQIP data sets. Aesthet Surg J.

